# Thermo–Mechanical Behavior and Constitutive Modeling of In Situ TiB_2_/7050 Al Metal Matrix Composites Over Wide Temperature and Strain Rate Ranges

**DOI:** 10.3390/ma12081212

**Published:** 2019-04-13

**Authors:** Kunyang Lin, Wenhu Wang, Ruisong Jiang, Yifeng Xiong, Chenwei Shan

**Affiliations:** 1 School of Mechanical Engineering, Northwestern Polytechnical University, Xi’an 710072, China; linkunyang@mail.nwpu.edu.cn (K.L.); npuwwh@nwpu.edu.cn (W.W.); xiongyifeng@mail.nwpu.edu.cn (Y.X.); shancw@nwpu.edu.cn (C.S.); 2 School of Aeronautics and Astronautics, Sichuan University, Chengdu 610065, China

**Keywords:** constitutive model, flow stress, metal matrix composites, TiB_2_ particle

## Abstract

The thermo–mechanical behavior of in situ TiB_2_/7050 Al metal matrix composites is investigated by quasi-static and Split Hopkinson Pressure Bar compression tests over a wide range of temperature (20~30 °C) and strain rate (0.001~5000 s^−1^). Johnson–Cook and Khan–Liu constitutive models determined from curve fitting and constrained optimization are used to predict the flow stress during deformation. In addition, another Johnson–Cook model calculated from an orthogonal cutting experiment and finite element simulation is also compared in this study. The prediction capability of these models is compared in terms of correlation coefficient and average absolute error. Due to the assumptions in orthogonal cutting theory, the determined Johnson–Cook model from cutting cannot describe the material deformation behavior accurately. The results also show that the Khan–Liu model has better performance in characterizing the material’s thermo–mechanical behavior.

## 1. Introduction

Over the past decades, particle reinforced metal matrix composites (PRMMCs) have received wide attention against conventional material in the structural engineering field due to their excellent properties, such as high strength-to-weight ratio, high modulus-to-weight ratio and excellent fatigue resistance [1,2,3,4]. Severe processing technologies have been used to fabricate PRMMCs with different kinds of reinforcement particulates [5,6,7]. As classified by the forming method of reinforced particles, the PRMMCs can be classified into two kinds: in-situ and ex-situ PRMMCs. Ex-situ PRMMCs means that the reinforced particles are added in the matrix in molten or powder form by physical mixing or other ways. For the in-situ PRMMCs, the reinforced particles are formed in the metallic matrix by a chemical reaction between different reaction salts under certain conditions. Contributed by the chemical reaction, in-situ PRMMCs always have cleaner particulate–matrix interfaces, little particulate size and more uniform particle distribution than ex-situ PRMMCs. Therefore, the in-situ PRMMCs have shown better mechanical properties. Although great developments have been made in the processing technology of PRMMCs, a systematic study on the response of PRMMCs over high strain rate and high temperature is scare. Thus, for the widespread use and numerical simulation technology of PRMMCs, it is necessary to characterize the stress–strain behavior of PRMMCs over a wide range of strain rate and temperature.

Constitutive models are mathematical representations describing the stress–strain behavior of a material when it is subject to loading under different strain rates and temperatures [8]. An ideal constitutive model should have the capability to precisely describe the strain hardening effect, strain rate effect and thermal softening effect. The commonly used constitutive equation can be categories as phenomenological based constitutive model and physical based constitutive model. Physical based constitutive models are rooted on the micromechanism of the crystal plastic deformation and predict the mechanical behavior of the material by introducing the concepts of thermal activation energy and dislocation interaction, as well as dislocation density evolution mechanisms [9]. These kind of models are always established under physical assumptions with a large number of material constants. For the PRMMCs, the existence of brittle particles and ductile matrix complicates the plastic deformation process under quasi-static or dynamic conditions. Four types of strengthening mechanisms in PRMMCs (Hall–Petch strengthening, Orowan strengthening, coefficient of thermal expansion mismatch strengthening and elastic modulus mismatch strengthening) and the particle cluster effect aggravate the process of physical modeling of PRMMCs [10]. Especially, it is difficult to conduct the numerical simulation by physical-based constitutive model. Therefore, a phenomenological model is always preferred for engineering applications.

The normally used phenomenological constitutive models are Johnson–Cook (JC) model [11], the Norton–Hoff law [12,13], Zerilli–Armstrong (ZA) model [14], KHL model [15] and Cowper–Symonds model [16]. Due to the convenience form with just five material constants, JC model has always been used to describe the flow stress behavior of different materials and has been widely combined into many commercial software for numerical simulation. Zhou et al. [17] developed a 2D orthogonal cutting simulation by JC model to study the formation mechanism of edge defects while machining SiCp/Al composites. The disadvantage of JC model is that the strain hardening, strain rate hardening as well as thermal softening effect of a material are multiplied together without considering the coupled effect which has been shown on many materials. In order to address this issue, many modified JC models have been proposed. Song et al. [18] modified the power law hardening term in the JC model by a quadratic function to strain and substituted the thermal term by an exponential function considering the coupled effects of temperature and strain rate. A good agreement between the model prediction and the result of the Hopkinson tension bar experiment for the mechanical properties of TiCp/Ti metal matrix composites in the temperature range of 20 °C to 650 °C and strain rate between 10^−3^ and 10^3^ s^−1^ demonstrated the reliability of the modified JC model. Rudra et al. [19] used modified JC and ZA models to describe the flow stress of SiC/Al5083 composites and found the modified ZA model exhibited a higher prediction accuracy. Another typical phenomenological model is the KHL model and the modified KHL model [9,15,20,21,22,23]. The KHL model has some coupled effects on the description of work hardening of a material. From the research of Xu and Huang [24] on the thermomechanical behavior of tungsten-based composites, they found that the KHL model had a better description ability than JC model for both quasi-static and dynamic experiment data. This was consistent with the conclusions by Khan and Liang [15].

In the manufacturing field, specified cutting experiments are also utilized to determine the material constants in the constitutive model. Tounsi et al. [25] proposed a methodology to identify the five material constants in the JC model using the basic mechanics of orthogonal cutting process in conjunction with orthogonal cutting experiments. It is well known that an accurate constitutive model is a precondition for the simulation of cutting process. However, the constitutive model of a material usually has different material constants combination form different researches. Umbrello et al. [26] compared five different sets of material constants in the JC model for AISI 316 L steel from studies on the finite element simulation of orthogonal cutting. They found that the material constant in the constitutive model had a sensitive effect on the simulation accuracy. Ducobu et al. [27] collected twenty sets of JC constitutive model for Ti6Al4V from the literature and found that the material constants obtained by different authors varied dramatically. Moreover, Daoud et al. [28] found that the material constants of a JC model, determined through orthogonal cutting tests by cutting tools with different rake angles, had different values, resulting in different simulation results. Thus, it is necessary to evaluate the difference of constitutive models determined from an orthogonal cutting process and conventional loading test (quasi static and dynamic). On the other hand, although great developments have been made in the constitutive model of different materials, a systematic research on the constitutive model of PRMMCs under different strain rates and temperatures is limited. This will also hinder the finite element simulation technology of PRMMCs.

In this research, the flow stress behavior of TiB_2_/7050 Al composites is studied. There are two aims of this paper. The first is to systematically study the thermo–mechanical properties and deformation mechanism of TiB_2_/7050 Al composites. The other is to study the descriptive ability of JC and KL models concerning TiB_2_/7050 Al composites. In addition, the result of the JC model obtained by an orthogonal cutting experiment from our previous research [29] is compared. The established constitutive model can be well used for the simulation study of TiB_2_/7050 Al composites under different processing technologies. The structure of this paper is organized as below. In Section 2, the procedure of quasi-static and a dynamic compressive experiment is presented. The experimental results are shown in Section 3. In Section 4, the JC and KL models are established based on the experiment results. The reliabilities of these models are evaluated in Section 5. Finally, the conclusions are given in Section 6.

## 2. Experimental Materials and Procedures

### 2.1. Materials

**Figure 1:**
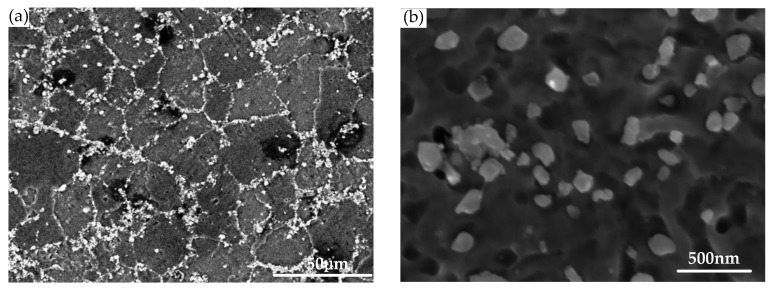
Microstructure of TiB_2_/7050 Al composite: (**a**) SEM images shows the distribution of TiB_2_ particles; (**b**) Magnified TiB_2_ particles aggregated along the grain boundaries.

The in-situ TiB_2_ (6 wt%) particle-reinforced 7050 aluminum matrix composites (shortened as TiB_2_/7050 Al composites) used in this study are the same as those in a previous work [29]. The material was fabricated via the controllable salt-metal reaction technique of K_2_TiF_6_ and KBF_4_ by the State Key Laboratory of Metal Matrix Composites of China [3,30]. Table 1 presents the nominal chemical composition of TiB_2_/7050 Al composite. Contributed by the in-situ synthesis method, the TiB_2_ particles are distributed uniformly in the matrix with a fine size that ranged from 20 to 500 nm [31]. Typical microstructure images of TiB_2_/7050 Al composites by SEM are shown in Figure 1.

The cylindrical specimens were turned by a CNC lathe. Each specimen has a size of 4 mm in length and 5 mm in diameter. The specimens were polished on waterproof abrasive paper with fine grit mesh to reduce end friction during the quasi-static and dynamic compression experiments.

### 2.2. Quasi-Static Uniaxial Compression Experiments

**Figure 2:**
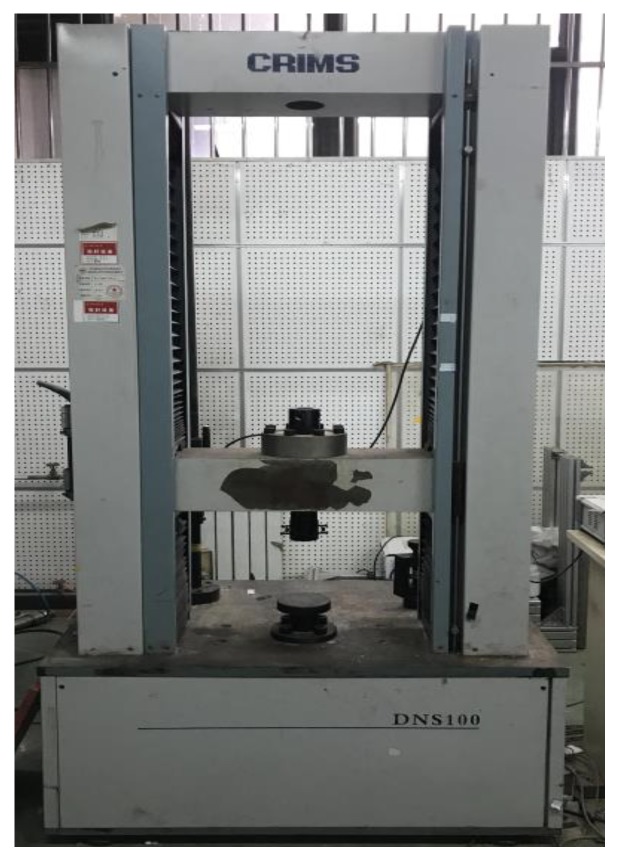
DNS100 electromechanical testing machine.

Quasi-static experiments were conducted using a DNS100 electromechanical testing machine (as shown in Figure 2) at a strain rate of 10^−3^ s^−1^ under a temperature range of 20, 100, and 200 °C. The high temperature experiments were performed with a radiant-heating furnace. The inside temperature was measured by an artificial thermocouple arrangement. Before each experiment, the specimen was kept warm for 5 min to make sure that there was uniform heat.

### 2.3. Dynamic Compressive Experiments

**Figure 3:**
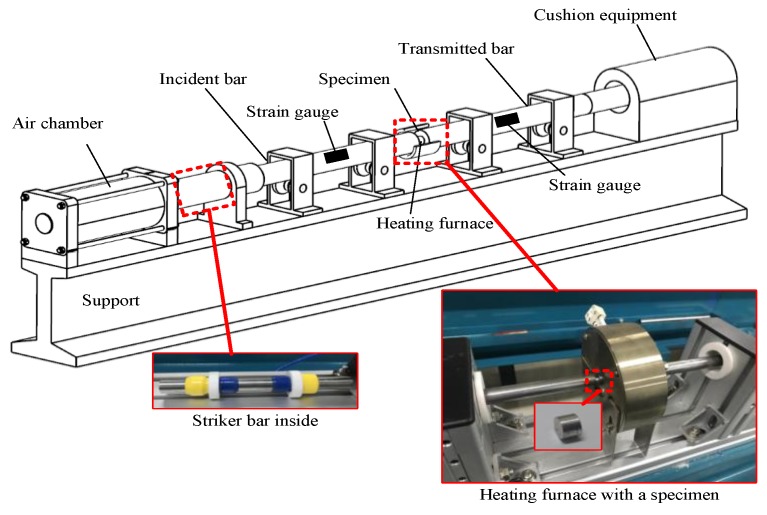
The diagram illustration of SHPB system.

Dynamic compressive experiments were performed using the compression Split Hopkinson Pressure Bar (SHPB) technique as shown in Figure 3. The dynamic experiments were conducted under the temperature range of 20, 100, and 200 °C, and the strain rate range of 1000, 3000, and 5000 s^−1^. The SHPB bars were manufactured by a steel with an elastic modulus of 210 GPa. The bars were 19 mm in diameter. During the experiments, specimens were placed between the incident bar and the transmitted bar. The interface between the specimen and the two bars was lubricated by molybdenum powder to reduce end friction and guarantee a uniform stress state during the experiment. A shaper made from copper was used to reduce wave oscillation during each test. The waves were measured by a SDY2107B ultrahigh dynamic strain indictor. The radiant heating furnace used in the quasi-static experiment was also used in the dynamic compressive experiment with a 5-min holding time.

## 3. Experiment Results

**Figure 4:**
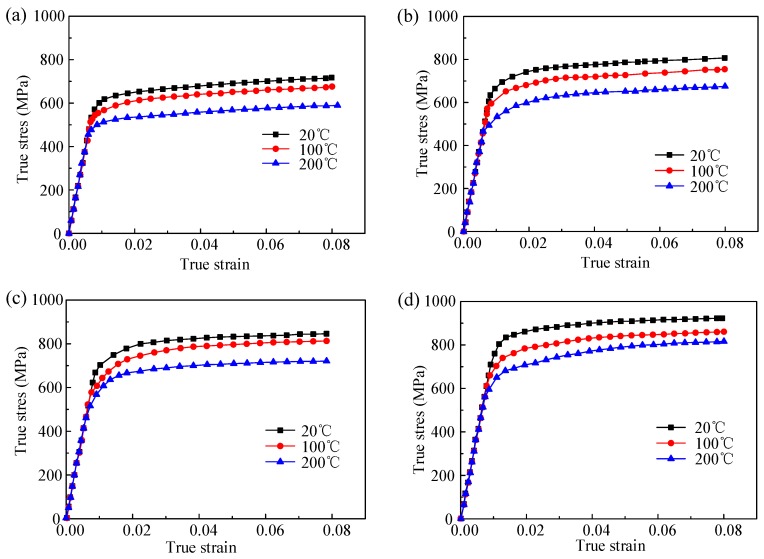
True stress–strain curves for TiB_2_/7050 composites under different temperature with the strain rate of (**a**) 0.001 s^−1^, (**b**) 1000 s^−1^, (**c**) 3000 s^−1^, and (**d**) 5000 s^−1^.

The experimental results of true strain versus true stress from the uniaxial and dynamic compressive experiment over different strain rate (10^−3^ s^−1^, 1000 s^−1^, 300 s^−1^, 5000 s^−1^) and temperature (20 °C, 100 °C, 200 °C) are shown in Figure 4. The strain rate and temperature have profound effect on the flow stress behavior. It can be seen that in the strain rate of 10^−3^ s^−1^ under room temperature, the strength of TiB_2_/7050 Al composites is higher than that of aluminum alloy. This has been demonstrated that in metal matrix composites, the mismatch of thermal expansion coefficient and elastic modulus of matrix and reinforcement results in the geometrically dislocations around particles and contributes to work hardening effect of metal matrix composites. The phenomenon is known as Orowan strengthening effect. As the increase of temperature, the flow stress decreases at a specified strain rate due to the soften effect. On the other hand, the flow stress increases with the larger strain rate for a specified temperature. However, under each condition, the flow stress increases rapidly with the increase of plastic strain under 0.02 and the flow stress tends to reach a plateau state with the plastic strain larger than 0.02. This is due to the decrease of work hardening rate. The increase of flow stress is due to strain hardening. However, the degree of dynamic recovery increases with higher strain. It seems that a nearly balance state is achieved with the increasing plastic strain.

## 4. Constitutive Modeling

It is well known that the strain rate, temperature, and plastic strain have significant effects on the plastic behavior of metal material. In order to predict the flow stress and to describe the strain rate effect, temperature effect, and work hardening effect, different constitutive models have been developed by researchers in different domains [32]. In this section, the JC and KL models are used to describe the plastic behavior of TiB_2_/7050 composites. In our previous work, the material constants in the JC model are obtained from basic mechanics of orthogonal cutting. The prediction ability of the JC model from orthogonal cutting experiment (shorted as JC model from cutting) will be compared with the result determined by the compressive experiment in this study. The brief procedures of parameter determination are introduced in this section. In order to compare the description capability of different models, correlation coefficient (*R*) and average absolute error (∆_ave_) are calculated to evaluate the deviation:

(1) $$\Delta_{ave}=\frac{1}{N}\sum_{i=1}^{N}\left|\frac{\sigma_{\exp}^{i}-\sigma_{\mathrm{mod}\mathrm{el}}^{i}}{\sigma_{\exp}^{i}}\right|\times 100$$

(2) $$R=\frac{\sum_{i=1}^{N}\left(\sigma_{\exp}^{i}-{\bar{\sigma }}_{\exp})(\sigma_{\mathrm{mod}\mathrm{el}}^{i}-{\bar{\sigma }}_{\mathrm{mod}\mathrm{el}}\right)}{\sqrt{\sum_{i=1}^{N}{\left(\sigma_{\exp}^{i}-{\bar{\sigma }}_{\exp}\right)}^{2}\sum_{i=1}^{N}{\left(\sigma_{\mathrm{mo}\mathrm{del}}^{i}-{\bar{\sigma }}_{\mathrm{model}}\right)}^{2}}}$$

where *σ*_exp_ and *σ*_model_ are flow stress obtained by experiment and prediction model, respectively. ${\bar{\sigma }}_{\exp}$ and ${\bar{\sigma }}_{\mathrm{model}}$ are the mean experimental and model calculated values, respectively.

### 4.1. Johnson–Cook Constitutive Model

The Johnson–Cook (JC) model [11] is expressed as follows:

(3) $$\sigma =(A+B\varepsilon^{n})(1+C\ln{\dot{\varepsilon }}^{*})(1-T^{*}{}^{m})$$

where *σ* is the flow stress, *ε* is equivalent plastic strain, ${\dot{\varepsilon }}^{*}=\dot{\varepsilon }/{\dot{\varepsilon }}_{0}$ is the plastic strain rate in which $\dot{\varepsilon }$ is the current plastic strain and $\dot{\varepsilon_{0}}$ is the reference strain rate (10^−3^ s^−1^), $T^{*}=(T-T_{r})/(T_{m}-T_{r})$ in which T is the current temperature, *T*_r_ is the reference temperature (20 °C), and *T*_m_ is melt temperature (476 °C) of the material, and *A*, *B*, *c*, *m*, *n* are all material constants.

The material constants in the JC model can be determined progressively from the experiment result by the following steps:

(1) By using the experiment result at reference strain rate and temperature, Equation (1) reduces to:
  (4) $$\sigma =A+B\varepsilon^{n}$$
  (5) ln(σ−A)=lnB+nlnε
  The material constant *A* can be got from the yield stress when plastic strain *ε* = 0. By the stress data at different plastic strains, *B* and *n* can be determined from the intercept and slope of the ln(*σ*-*A*) versus ln*ε* fitting line respectively.
(2) At the reference temperature, the third bracket in Equation (1) become unity and the Equation (1) reduces to:
  (6) $$\sigma =(A+B\varepsilon^{n})(1+C\;\ln{\dot{\varepsilon }}^{*})$$
  (7) $$\frac{\sigma }{\left(A+B\varepsilon^{n}\right)}-1=C\ln{\dot{\varepsilon }}^{*}$$
  By selecting a series of plastic strain at different strain rates, the material constant *C* can be determined from the above relationship in Equation (7).
(3) At the reference strain rate, the second bracket in Equation (3) become unity and the Equation (3) becomes:
  (8) $$\sigma =(A+B\varepsilon^{n})(1-T^{*}{}^{m})$$
  (9) $$\ln\left(1-\frac{\sigma }{\left(A+B\varepsilon^{n}\right)}\right)=m\ln T^{*}$$
  By selecting a series of plastic strain at different temperatures, the material constant m can be determined from the above relationship.

**Figure 5:**
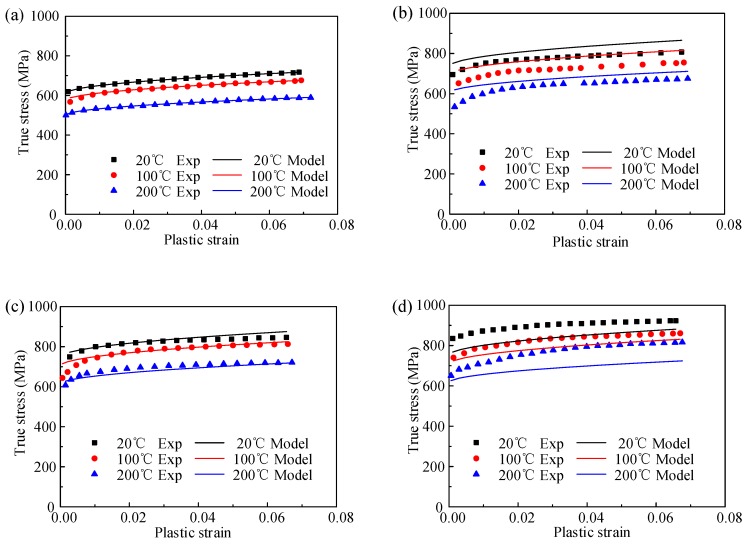
Comparison between experiment result and the predicted flow stress by JC model at different strain rate of (**a**) 0.001 s^−1^, (**b**)1000 s^−1^, (**c**) 3000 s^−1^, and (**d**) 5000 s^−1^.

Finally, in order to obtain a globally optimal solution, a least-squared-based optimization program is used for the calculation. The five material constants from the above steps are set as an initial value in the optimization program. The optimized values are listed in Table 2. The comparison between experimental data and predicted data by the JC model is shown in Figure 5. The correlation coefficient (*R*) and average absolute error (∆_ave_) of the JC model are 91.1% and 4.11%, respectively.

### 4.2. Johnson–Cook Constitutive Model Obtained from Cutting Experiment

**Figure 6:**
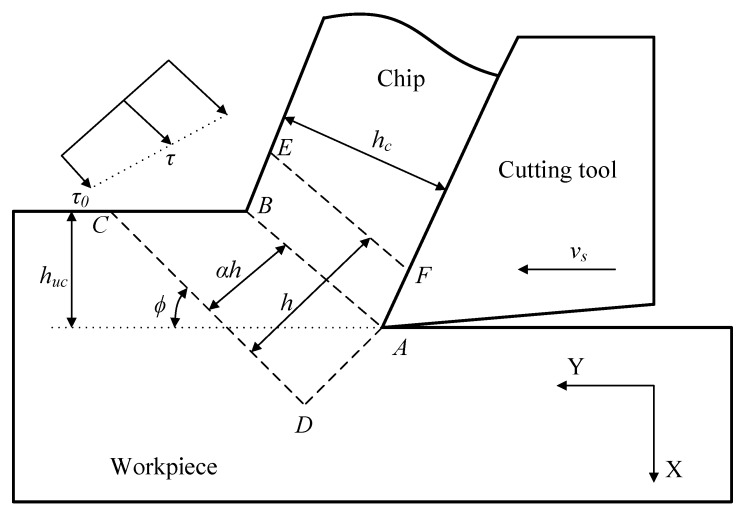
The diagram illustration of orthogonal cutting experiment.

In our previous study [29], the JC constitutive model of TiB_2_/7050 Al composites is calculated from the basic mechanics of orthogonal cutting and finite element simulation based on reference [25]. A set of orthogonal cutting experiments were conducted by a CNC lathe. During the cutting process, the cutting forces were measured by a Kistler 9257B three-component piezoelectric dynamometer. After each test, the cutting chips were collected and further analyzed for the shear stress, strain, strain rate and temperature in the shear zone. A more detailed description of orthogonal cutting experiment with cutting parameter ranges can be found in the reference [29]. The basic schematic diagram of orthogonal cutting experiment is shown in Figure 6. The physical quantities on the shear plane are determined based on the following formulas:

(10) $$\sigma =\sqrt{3}\left|\tau \right|$$

(11) $$\tau =\frac{\sin\phi }{wh_{uc}}(F_{Y}\cos\phi -F_{X}\sin\phi )$$

(12) $$\dot{\varepsilon }=\frac{2v_{s}\cos\gamma }{\sqrt{3}h\cos(\phi -\gamma )}$$

(13) $$\varepsilon =\frac{\cos\gamma }{\sqrt{3}\cos(\phi -\gamma )\sin\phi }\left(\frac{1}{2}+\frac{\cos(2\phi -\gamma )}{2\cos\gamma }\right)$$

(14) $$T=T_{0}-\left[\left(\frac{1}{2}+\frac{\cos(2\phi -\gamma )}{2\cos\gamma }\right)\frac{\cos\gamma }{\rho C_{p}\cos(\phi -\gamma )\sin\phi }\right]\left[\frac{2\tau +\tau_{0}}{3}\right]$$

where *F*_X_ is the measured cutting force component along the X direction, *F*_Y_ is the measured cutting force components along the Y direction, *γ* is the tool rake angle, *Φ* is the shear angle, *h* is the primary shear zone thickness, *h*_uc_ is uncut chip thickness, *h* is the chip thickness, *v*_s_ is the cutting speed, *α* is the proportion of the main shear zone, *σ* is the flow stress, *ε* is the effective strain, $\dot{\varepsilon }$ is the effective strain rate, *τ* is the shear stress in the shear plane, *τ*_0_ is the shear stress in the main shear zone inlet CD, *T* is the temperature on the shear plane, *T*_0_ is room temperature, *ρ* is the density of material, and *C_p_* is the specific heat. The other parameters have the same definition with the JC model in Equation (3). Then, these quantities are applied to determine the material constants in the JC model by the following Equation (15) using a genetic algorithm:

(15) $$f(A,B,C,m,n)=\min\left\{{\left\|\frac{A+B\varepsilon^{n}}{\sqrt{3}\left|\tau \right|}\left[1+C\ln\left(\frac{\dot{\varepsilon }}{{\dot{\varepsilon }}_{0}}\right)\right]\left[1-{\left(\frac{T-T_{r}}{T_{m}-T_{r}}\right)}^{m}\right]-1\right\|}_{\infty }\right\}$$

Finally, the material constants of the JC model are obtained by matching the FEM simulation with the orthogonal cutting experiment under the same cutting condition. The obtained material constants are shown in the Table 3.

**Figure 7:**
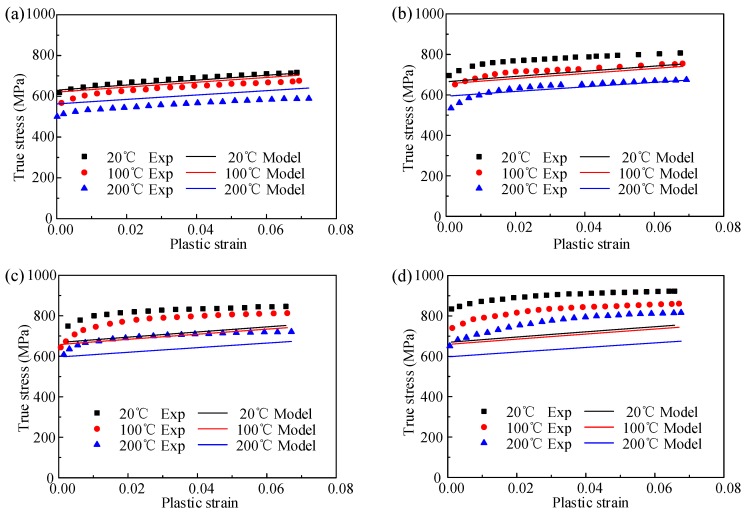
Comparison between experiment result and the predicted flow stress by JC model for orthogonal cutting experiment at different strain rate of (**a**) 0.001 s^−1^, (**b**)1000 s^−1^, (**c**) 3000 s^−1^, and (**d**) 5000 s^−1^.

The comparison between the experiment data and predicted date by the JC model from an orthogonal cutting experiment is shown in Figure 7. The correlation coefficient (*R*) and average absolute error (∆_ave_) of the JC model from the cutting experiment are 78.35% and 9.23%, respectively.

### 4.3. Khan–Liu Constitutive Model

The Khan–Liu (KL) constitutive model [9] is expressed as follows:

(16) $$\sigma =\left[A+Be^{-C_{3}\frac{\dot{\varepsilon }}{{\dot{\varepsilon }}_{0}}}{\left(\frac{T_{m}-T_{r}}{T_{m}-T}\right)}^{m_{3}}\varepsilon^{n_{0}}\right]e^{C_{1}\frac{\dot{\varepsilon }}{{\dot{\varepsilon }}_{0}}}{\left[\frac{T_{m}-T_{r}}{T_{m}-T}\right]}^{m_{2}}$$

where $\dot{\varepsilon }$ and $\dot{\varepsilon_{0}}$ are the current strain rate and reference strain rate. *A*, *B*, *C*_3_, *m*_3_, *n*_0_, *C*_1_, *m*_2_ are material constants, and the other parameters have the same definition with the JC constitutive model. The steps on the determination of material constants in the KL model are described briefly below and the details can be found in reference [10]:

(1) The material constant *A* can be determined from the yield stress when the current strain rate $\dot{\varepsilon }$ = ${\dot{\varepsilon }}_{r}$, current temperature *T* = *T*_r_ and plastic strain *ε* = 0.
(2) By using the experimental result at a reference temperature and *ε* = 0, the yield stress *σ*_Y_ at different conditions can be obtained. Equation (16) reduces to:
  (17) $$\sigma_{Y}=Ae^{C_{1}\frac{\dot{\varepsilon }}{{\dot{\varepsilon }}_{0}}}$$
  (18) $$\ln\frac{\sigma_{Y}}{A}=C_{1}\frac{\dot{\varepsilon }}{{\dot{\varepsilon }}_{0}}$$
  The material constant *C*_1_ can be evaluated from the slope of $\ln\frac{\sigma_{Y}}{A}$ versus $\frac{\dot{\varepsilon }}{{\dot{\varepsilon }}_{0}}$.
(3) By using the yield stress *σ_Y_* of the experiment result when the strain rate $\dot{\varepsilon }$ = ${\dot{\varepsilon }}_{0}$, Equation (16) reduces to:
  (19) $$\sigma_{Y}=A{\left(\frac{T_{m}-T_{r}}{T_{m}-T}\right)}^{m_{2}}$$
  (20) $$\ln\left(\frac{\sigma_{Y}}{A}\right)=m_{2}\ln(\frac{T_{m}-T_{r}}{T_{m}-T})$$
  The material constant *m*_2_ can be determined from relationship between $\ln(\frac{\sigma_{Y}}{A})$ and $\ln\left(\frac{T_{m}-T}{T_{m}-T_{r}}\right)$ using the yield stress at different temperature.
(4) By using the experiment result at reference strain rate and temperature, Equation (16) reduces to:
  (21) $$\sigma =A+B\varepsilon^{n}$$
  (22) ln(σ−A)=lnB+nlnε
  The material constant *A* can be obtained from the yield stress when plastic strain *ε* = 0. By the stress data at different plastic strains, *B* and *n* can be determined from the intercept and slope of the ln(*σ*−*A*) versus ln*ε* fitting line, respectively.
(5) When strain rate $\dot{\varepsilon }$ = 1, Equation (16) reduces to:
  (23) $$\sigma =\left[A+B{\left(\frac{T_{m}-T}{T_{m}-T_{r}}\right)}^{m_{3}}\varepsilon^{n}\right]{\left[\frac{T_{m}-T}{T_{m}-T_{r}}\right]}^{m_{2}}$$
  (24) $$\ln\left[\frac{\sigma }{{\left(\frac{T_{m}-T}{T_{m}-T_{r}}\right)}^{m_{2}}}-A\right]=\ln(B\varepsilon^{n_{0}})+m_{3}\ln\left(\frac{T_{m}-T}{T_{m}-T_{r}}\right)$$
  Then the material constant *m*_3_ can be determined from the above relationship by the stress–strain data at various temperatures and plastic strains.
(6) When the current temperature *T* = *T*_r_, Equation (16) reduces to:
  (25) $$\sigma =(A+Be^{-C_{3}\frac{\dot{\varepsilon }}{{\dot{\varepsilon }}_{0}}}\varepsilon^{n_{0}})e^{C_{1}\frac{\dot{\varepsilon }}{{\dot{\varepsilon }}_{0}}}$$
  (26) $$\ln\left(\frac{\frac{\sigma }{e^{C_{1}\frac{\dot{\varepsilon }}{{\dot{\varepsilon }}_{0}}}}-A}{B\varepsilon^{n_{0}}}\right)=-C_{3}\frac{\dot{\varepsilon }}{{\dot{\varepsilon }}_{0}}$$
  Then the material constant *C*_3_ can be determined from the above relationship by the stress–strain date at different strain rate and plastic strain.

**Figure 8:**
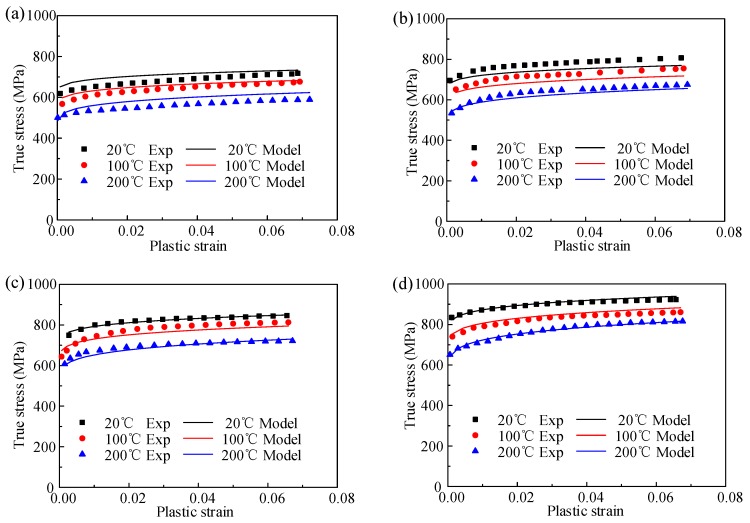
Comparison between experiment result and the predicted flow stress by KL model at different strain rate of (**a**) 0.001 s^−1^, (**b**)1000 s^−1^, (**c**) 3000 s^−1^, and (**d**) 5000 s^−1^.

Finally, in order to obtain the globally optimal solution, a least-squared-based optimization program is used for the calculation. The seven-material constants from the above steps are set as initial values in the optimization program. The optimized values obtained by the program are listed in Table 4. The comparison between experimental data and predicted data by KL model is shown in Figure 8. The correlation coefficient (*R*) and average absolute error (∆_ave_) of KL model are 97.68% and 2.61%, respectively.

## 5. Comparison of the Constitutive Models

**Figure 9:**
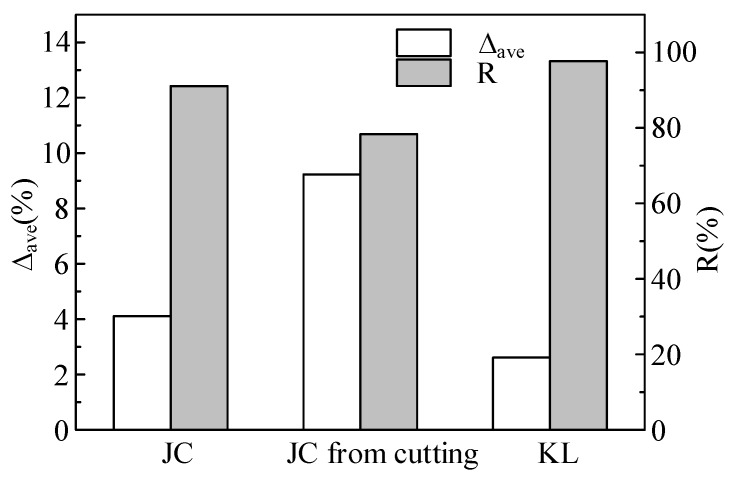
Comparison of correlation coefficient (R) and average absolute error (∆_ave_) of different models in prediction of experiment data.

The comparisons of correlation coefficient (*R*) and average absolute error (∆_ave_) of different models in the prediction of experimental data are shown in Figure 9. It can be seen that the KL mode has the lowest prediction error and highest correlation coefficient for fitting the experimental data. This is due to the fact that the KL model has shown good performance in characterizing thermal softening of materials at a high strain rate.

The JC model has been widely used in a number of commercial simulation software for predicting the flow stress behavior of different materials. However, the performance of the JC model for predicting the flow stress behavior of TiB_2_/7050 Al composites is worse than the KL model. From Figure 5, the prediction results of the JC model only agree well with the experiment data under the reference strain and reference temperature conditions. The phenomenon is consistent with the research by Lin et al. [33] while testing quasi tensile behavior of alloy steel and Song et al. [18] while testing dynamic tensile behavior of TiC_p_/Ti composites. They both found that the prediction error of the JC model becomes larger with the test strain rate and temperature far away from reference conditions. The result is due to the fact that the JC model multiplied the strain hardening effect, strain rate hardening effect and softening effect together without considering the coupled effect among them. However, the coupled effect has been demonstrated by many researchers in different materials [8].

For the JC model from cutting, the prediction error is much larger than the other two. The prediction result of the JC model from cutting in Figure 7 shows a nearly linear relationship between stress and strain without obvious yield stages. The relationship is not in accordance with the real physical deformation process. This is due to the fact that the calculation theory of constitutive model from orthogonal cutting is based on many assumptions, such as plane strain conditions, sharp cutting edge, and constant thickness of primary shear zone and so on. In addition, the geometrical shape of the cutting tool has significant effect on the cutting process, which induces different material constants combinations. Although the material constants determined from the orthogonal cutting experiment can be used for accurate prediction of the cutting force and chip morphology, the result cannot describe the flow stress during material deformation exactly.

**Figure 10:**
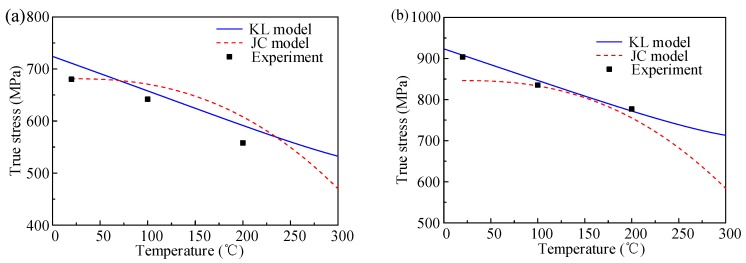
Comparison of temperature effect by JC and KL model at plastic strain 0.03 for different strain rate: (**a**) 0.001 s^−1^, and (**b**) 5000 s^−1^.

The comparison of the temperature effect by the JC and KL models at plastic strain of 0.03 for the strain of 0.001 s^−1^ and 5000 s^−1^ is shown in Figure 10. When the strain rate is 0.001 s^−1^, the JC model predicts the true stress exactly at the reference temperature of 20 °C, but the KL model predicts a higher value at this temperature. At the temperatures of 100 °C and 200 °C, both the JC and KL models overestimate the true stress at a 0.001 s^−1^ strain rate. For 5000 s^−1^ in Figure 7b, it can be seen clearly that the KL model shows good correlation with the experiment result. The JC model gives a lower prediction value at 5000 s^−1^.

**Figure 11:**
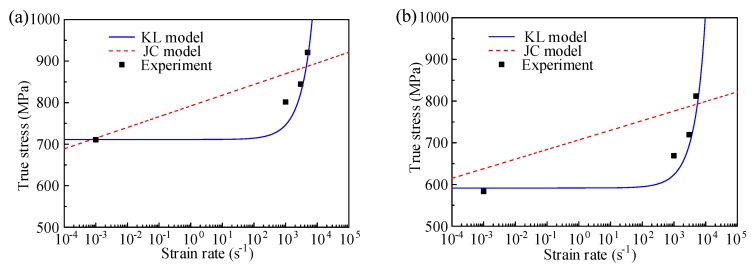
Comparison of strain rate effect by JC and KL model at plastic strain 0.06 for different temperature: (**a**) 20 °C, and (**b**) 200 °C.

Figure 11 illustrates the strain effect described by the JC and KL model at a plastic strain of 0.06, for the temperature of 20 °C and 200 °C. As the quasi-static compressive test was conducted only at the strain rate of 10^−3^, the experimental data at a quasi-static rage is limited. It is evident that the description ability of the strain rate effect by the KL model is better than the JC model.

Under different temperatures in Figure 11, the strain rate effect described by the JC model is nearly a linear relationship, which is not consistent with the practice. This is due to the defect of the JC model by describing a linear increase of flow stress with log strain rate. For the KL model, the coupled effect of strain rate and temperature is considered, which is in agreement with the experimental result. Clearly, in a quasi-static state, the effect of strain rate on the true stress is inconspicuous. However, for the strain rate that is larger than 1000 s^−1^, the growth trend of true stress is significant with higher strain rate. The strain rate effect under ultra-high strain rate needs to be demonstrated in a future study. Therefore, in the studied conditions, the KL model presents a better performance for characterizing the thermo–mechanical behavior of TiB_2_/7050 Al composites.

## 6. Conclusions

The thermo–mechanical behavior of TiB_2_/7050 Al composites is investigated over temperatures ranging from 20 °C to 200 °C and strain rates ranging from 10^−3^ s^−1^ to 5000 s^−1^ in this study. The JC and KL constitutive models are used to predict the flow stress behavior. The JC model obtained from the orthogonal cutting experiment in our previous research is referenced for comparison. Correlation coefficient and average absolute error are calculated to evaluate the prediction ability of these three models. The capabilities of the JC and KL constitutive models in a characterizing temperature effect and strain effect of TiB_2_/7050 Al composites are also discussed. The main conclusions are drawn as follows:

(1) The strain rate and temperature have profound effects on the flow stress behavior of TiB_2_/7050 Al composites. As the increase of temperature, the flow stress decreases at a specified strain rate due to the soften effect. On the other hand, the flow stress increases with the larger strain rate for a specified temperature.
(2) Due to the mismatch of thermal expansion coefficient and elastic modulus of aluminum matrix and TiB_2_ reinforcement particle, geometrically dislocation occurs around the particles and contributes to the work hardening effect of TiB_2_/7050 Al composites. The strength of TiB_2_/7050 Al composites is much larger than the aluminum matrix.
(3) Compared with the JC constitutive model, the KL constitutive model performs better to predict the stress strain behavior of TiB_2_/7050 Al composites as it has a lower average absolute error (2.61%) and higher correlation coefficient (97.68%) with the experiment result. In addition, the KL model has shown better performance in characterizing the temperature effect and strain effect than JC model.
(4) Although the JC model from an orthogonal experiment can be used to simulate the cutting process, it cannot describe the flow stress exactly during material deformation. For an accurate constitutive model of a material, the basic tensile or compression test is deemed necessary.

## Figures and Tables

**Table 1:** Nominal chemical composition of TiB_2_/7050 Al composite.

Elements	TiB_2_	Cu	Mg	Zn	Zr	Al
Content/wt%	6	2.2	2.3	6.3	0.11	Balance

**Table 2:** The optimized material constants of JC model.

*A*	*B*	*C*	*m*	*n*
594	446.4538	0.0157	1.364	0.4655

**Table 3:** The material constant of JC model obtained by orthogonal cutting experiment [29].

*A*	*B*	*C*	*m*	*n*
630	1127	0.004	2.4	0.972

**Table 4:** The optimized material constants of KL model.

*A*	*B*	*C*_1_	*C*_3_	*n*_0_	*m*_2_	*m*_3_
602.6	235.5599	4.188 × 10^−8^	−4.2151 × 10^−8^	0.2211	0.6102	−1.2285
